# Graph theoretic network analysis reveals protein pathways underlying cell death following neurotropic viral infection

**DOI:** 10.1038/srep14438

**Published:** 2015-09-25

**Authors:** Sourish Ghosh, G. Vinodh Kumar, Anirban Basu, Arpan Banerjee

**Affiliations:** 1 National Brain Research Centre, NH 8, Manesar, Gurgaon -122051, Haryana, India

## Abstract

Complex protein networks underlie any cellular function. Certain proteins play a pivotal role in many network configurations, disruption of whose expression proves fatal to the cell. An efficient method to tease out such key proteins in a network is still unavailable. Here, we used graph-theoretic measures on protein-protein interaction data (interactome) to extract biophysically relevant information about individual protein regulation and network properties such as formation of function specific modules (sub-networks) of proteins. We took 5 major proteins that are involved in neuronal apoptosis post Chandipura Virus (CHPV) infection as seed proteins in a database to create a meta-network of immediately interacting proteins (1^st^ order network). Graph theoretic measures were employed to rank the proteins in terms of their connectivity and the degree upto which they can be organized into smaller modules (hubs). We repeated the analysis on 2^nd^ order interactome that includes proteins connected directly with proteins of 1^st^ order. FADD and Casp-3 were connected maximally to other proteins in both analyses, thus indicating their importance in neuronal apoptosis. Thus, our analysis provides a blueprint for the detection and validation of protein networks disrupted by viral infections.

Metabolic functions are outcomes of interactions among various cellular proteins. An emerging concept in the field of proteomics is that the understanding of these interactions is critical for elucidating the mechanism of metabolic functions^1^,^2^. However, parsing interactions important for certain functions or a disease involves analyzing huge interactomes containing information about a large number of genes and proteins along with their interacting partners. Mathematical modelling has been instrumental in analyzing these huge datasets and systematically understanding the interplay between various proteins and the metabolic functions involved^3^,^4^,^5^. Recent technical developments consider the huge protein interactome as a complex graph wherein individual proteins are nodes of the graph and the interactions are modelled as the edges^6^. Graph theoretic analysis provides an efficient handle to decipher various aspects of proteins in a network that interact with a specific functional objective. For example, is one protein more important than others, does a group of protein exhibit more interactions (densely connected) than other groups, do some proteins act as hubs through which majority of interactions are routed? Graph theory provides several parameters to study properties of constituent proteins in an interactome: degree centrality, clustering, betweenness, shortest path, modularity, etc., each of which may be meaningful for understanding function^7^,^8^,^9^. Based on a hypothesis about the operational structure of the interactome, researchers can decide upon what parameters to investigate.

Modularity quantifies how the nodes of a network are interacting among each other to form “hubs”^10^. Hubs or modules are closely interacting group of nodes with more connections within the module and sparse connections between modules. Real world networks such as the Internet, power grids, brain network exhibit such properties^11^,^12^,^13^. Thus, using modularity, researchers can quantify how many “hubs” of proteins are formed within a given interactome and whether a particular module is the key facilitator of a specific function/disease. Another useful measure using graph theory on interactome data is degree centrality. Degree centrality quantifies the individual contribution of a node (protein) to the interactome^14^. Depending upon the degree centrality score, the most dominating protein in a particular network can be characterized.

Chandipura Virus (CHPV) a member of the Rhabdoviridae family, has been ranked among the emerging viruses in the Indian subcontinent. CHPV was first identified in two patients in the year 1965 from the Chandipura village in Maharashtra (India)^15^. The first major outbreak took place in 2003 and resulted in death of 183 children. This was followed by sporadic attacks every year. Presently CHPV has a case-by-case fatality rate of around 55–77%^16^,^17^,^18^. The virus has been reported to cause encephalitis along with neurodegeneration leading to death. Common symptoms which have been diagnosed are high grade fever, vomiting, altered sensorium, generalized convulsions, decerebrate posture and coma. CHPV, being an arbovirus with sand flies (*Phlebotomus* sps.) as the carrier (vector), enters the host system through the skin, penetrating into the circulatory system of the body (which is also referred to as peripheral circulatory system). CHPV is cleared off the peripheral circulatory system by the host immune system within a couple of days post infection as observed in a mouse model^18^,^19^,^20^. But this virus finds a safe place to replicate in the brain.

In an earlier article some of us have shown in a mouse model that CHPV induces neuronal death through a Fas-mediated extrinsic apoptosis pathway^17^. From there we identified 5 proteins pertaining to the extrinsic apoptotic pathway. However, from this analysis we did not get the information about all the proteins that may be involved in the apoptotic process following CHPV infection.

In this article, we identified a large number of proteins (from an online database) that interact with the five proteins whose expressions were monitored in the earlier wet-lab experiment of CHPV infection^17^. The resultant network of proteins constituted a “1^st^ order interactome” Furthermore, we estimated a “2^nd^ order interactome” by identifying the proteins that were directly interacting with the 1st order interactome. We calculated the modularity of 1^st^ order and 2^nd^ order interactomes and degree centrality of individual proteins. These two measures quantified both a global measure of segregation of network and an individual connectivity measure of candidate proteins. 2^nd^ order connectome results were used to test the robustness of 1^st^ order connectome results and address the issue of predictive validity of the model. Together they revealed the protein-protein network configuration underlying neuronal apoptosis following CHPV infection. The issue of face validity was addressed by comparisons of empirical measures with those computed on simulated random networks. The methods and results obtained here provide an operational blueprint for understanding the pivotal dependencies of the virus within the host system and will help in the conceptualization and design of effective therapeutics.

## Results

**Figure 1:**
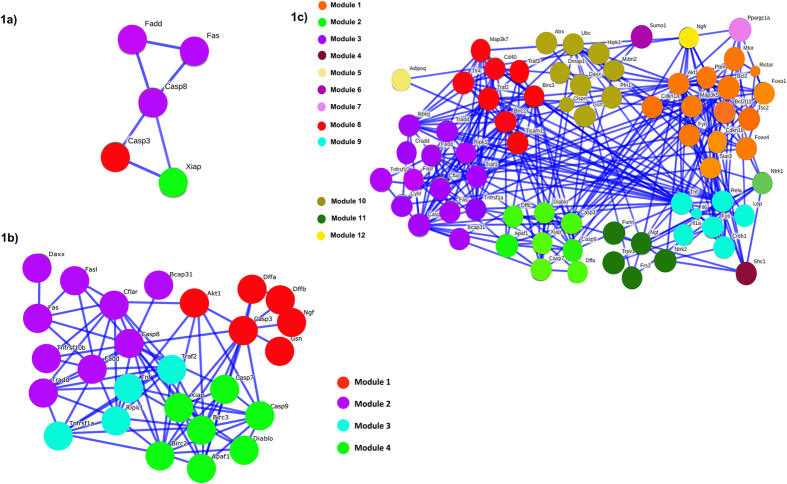
(**a**) Interactions between monitored proteins Fas, FADD, Casp-8, Casp-3 and XIAP estimated using STRING 9.1 database (**b**) Proteins interacting directly with Fas, FADD, Casp-8, Casp-3 and XIAP were estimated using STRING 9.1 database. The nodes represent the proteins while the lines indicate interactions in this 1^st^ order interactome. Only those proteins reported at a confidence level of 95% are considered. (**c**) The proteins interacting directly with the nodes of 1^st^ order interactome were extracted analogously to capture the 2^nd^ order interactome.

From our results in an earlier study^17^ we concluded that CHPV induces neuronal apoptosis through Fas-mediated extrinsic apoptotic pathway with the involvement of the following five proteins: Fas, Fas-associated Death Domain (FADD), Caspase-8 (Casp- 8), cleaved Caspase-3 (Casp- 3), and X-linked inhibitor of apoptosis (XIAP) (Fig. 1, see also Methods section for more details). These 5 proteins were inserted as inputs to STRING 9.1 online database (http://string-db.org/) for extraction of the 1^st^ and 2^nd^ order interactomes. The 1^st^ order interactome contained 26 proteins while the 2^nd^ order contained 71 proteins (Fig. 1b,c). The names of each protein from 1^st^ and 2^nd^ order interactomes are presented in Table 1.

The MATLAB-based Visual Connectome Toolbox^21^ was used for graph-theoretic analysis of 1^st^ and 2^nd^ order interactome data. We computed the degrees of freedom (degree centrality) for each protein in the 1^st^ & 2^nd^ order networks. Subsequently, we arranged them in a descending order (Table 2). From Table 2, we observed that in both 1^st^ and 2^nd^ order networks FADD and Casp-3 are the common members among the top 5 proteins having highest degree centrality values. Mutual cross-validation of results from 1^st^ and 2^nd^ order network analysis confirms that FADD and Casp-3 are dominant players in apoptotic pathway underlying CHPV infection in neurons. Modularity determines how well a network can be divided into subgroups (hubs). Generally the modularity score ranges between [−0.5, 1) with more modular networks having a positive score. A more randomly assigned network will have a modularity score of approximately zero. We computed modularity scores of both 1^st^ and 2^nd^ order networks sets. The modularity score of the 1^st^ order network was 0.36 while the 2^nd^ order was 0.41. Theoretically, due to the random partitioning of nodes into modules to initiate the graph theoretic algorithm, the results may vary trial to trial unless the modular structure is significantly unambiguous. In our data set the modularity score remained unchanged in all 50 repetitions of the analysis. Additionally, we evaluated the significance of the estimated modularity score by comparing with the modularity scores of a random network with an identical number of nodes. We start with an adjacency matrix with all values set to zero for a given number of nodes. Then we randomly assigned a value 1 in upper diagonal matrix locations. Finally, symmetric locations in lower diagonal positions are assigned values 1 to design the adjacency matrix for which network metrics are computed. Diagonal elements were always assigned a value 0 to avoid self-connections. The mean modularity score of a random network (50 repetitions) with 26 nodes was 0.13, whereas for a random network with 71 nodes, the score was 0.09. In both cases the estimated modularity values of the empirical networks were statistically significant at Bonferroni corrected p < 0.05 (*χ*^2^ = 20.67, *df* = 1 for 1^st^ order and *χ*^2^ = 58.40, *df* = 1 for 2^nd^ order interactome). In case of the 1^st^ order network, our analysis indicated the presence of 4 modules whereas in case of 2^nd^ order network 12 modules were identified.

**Figure 2:**
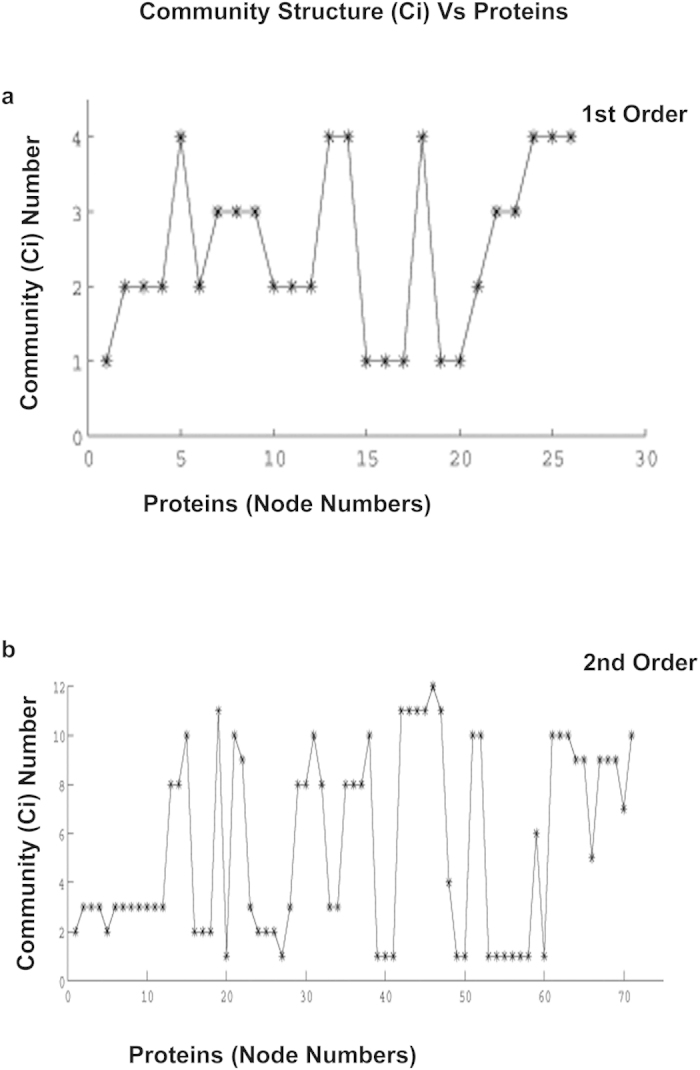
Representative plots for Community Structure (Ci) Vs protein node numbers were plotted in this figure for 1^st^ (**a**) and 2^nd^ (**b**) order interactomes. The Ci value from each analysis was obtained from running the codes for 50 times. Thereafter the mean Ci values corresponding to the mean Modularity score (Q) for each protein was plotted against the corresponding protein node number.

Using the Ci scores from Table 1 and Fig. 2, we color coded each module in Fig. 1(b,c). Modules 2 and 4 of the 1^st^ order interactome and module numbers 3 and 2 of 2^nd^ order interactome, respectively were presented in identical colors because they have multiple common members. The common members of module number 2 from 1st order and 3 from 2^nd^ order are Casp-8, Tnfrs10b, Cflar, Fas, FADD, TRADD. Module 4 from 1^st^ order and 2 from 2^nd^ order has Casp-9, Casp-7, XIAP, Apaf-1 and Diablo. Extraction of a consistent network structure from the analysis of 1^st^ order and 2^nd^ order interactomes provides confidence about the biological relevance of the key modules. Table 3 lists the UniProt IDs of all proteins identified in the 1^st^ and 2^nd^ order interactomes.

## Discussion

In this report we propose an analysis framework to compute the modular structure of a complex protein-protein interaction network (interactome). The choice of seed proteins: Fas, Fas associated Death Domain (FADD), Caspase-8, Caspase-3 and X-linked Inhibitor of Apoptosis Protein (XIAP) for the construction of the interactome was guided from our previous experimental findings^17^. These were apoptotic proteins over-expressed in mouse neurons following Chandipura Virus infection. We used the STRING 9.1 database to compute the first order interactome. There are currently several bioinformatics toolboxes available, each with their own set of unique controls. We chose STRING 9.1 because it was the only method to the best of our knowledge that allowed us to prune networks based on a statistical confidence level. However, it is pertinent to note that the database used to extract the interactome will immensely influence the estimation of any functional modular structure. A study comparing the interactomes extracted from several data sets may potentially help future research in terms of data interpretation. Next, we computed the graph theory metrics: Modularity Score (Q), Community Structure (Ci) and Degree Centrality (Z) to infer further about the protein-protein interactions underlying apoptosis. To establish the predictive validity of our analysis, we constructed a second order interactome based on secondary interacting partners of the seed proteins using the STRING 9.1 database (at 95% confidence) and re-calculated the graph theory metrics. The consistent presence of key protein assemblies in the first order and second order interactomes provides confidence regarding the robustness of our approach. Finally, we compared the closeness of modularity and degree centrality computed in empirical networks with that obtained for simulated random networks. Since, no modular structures are expected in a random network, this addressed the issue of face-validity, that is, whether the method is successful in extracting meaningful information and helped us control false positives.

Modularity score (Q) of a network ranges between [−0.5, 1) with negative Q scores signifying random interactions within the network. As the within group interactions increase, the network starts to become more modular and the Q value shifts more towards the positive side nearing to 1. For every network there exists an optimal Q value beyond which the modularity score cannot be enhanced even if we increase the number of modules. In our case we have determined the Q values of 1^st^ order and 2^nd^ order ineractome are 0.3911 and 0.4716, respectively. These scores were stable across 50 independent runs. The community structure also remained unchanged. These two findings give us the confidence to state that protein-protein interactions are indeed highly modular due to their inherent biological properties. Hence it is pertinent that the interactive nodes of both the networks have been classified into a maximum number of possible modules. Next we focus on each module to decipher their biological significance.

In the first order interactome, 4 interactive modules were identified (Fig. 1b). We could clearly characterize that all proteins segregated in separate modules on the basis of their functional role in the apoptotic process. Module 2 and 3 consist of all the proteins which are mostly known as death domain (DD) and death-inducing signalling complex (DISC). Proteins like FADD, TRADD, Cflar RIPK1, Daxx, Bcap31, Tnfrs1a & 10b have been reported to contribute the DD^22^,^23^ while Caspase 8 and FADD forms the DISC^24^,^25^. Other members like Fas (Module 2) and TNF (Module 3) are commonly known as the *initiators* of the death process. Module 3 consists of proteins that are co-stimulators of tumor necrosis factor (TNF) induced cell death whereas Module 2 consists of proteins that contribute to both Fas and TNF pathways. Module 4 is a heterogeneous group that consists of both apoptotic activators and inhibitors that belong to caspase group. XIAP has been previously reported both in our previous report and other researchers to be a Casp3 antagonist^17^ while Birc2 & Birc3 are well known to be in association with TNF to combat the apoptosis signalling^26^,^27^. Surprisingly, TNF and Birc2 and Birc3 were not in the same module in our 1^st^ order interactome. Other apoptotic activators of module 4 are Apaf-1 and Diablo along with the caspases like Casp 9 & 7. Overall this module represents proteins that are affecting the *intermediate* phase of apoptosis before the appearance of the final executioner of the apoptotic pathways. Module 1 is a classical cluster consisting of the close interactors of Casp3, the final *executioner* of the apoptotic pathway. This module consists of some of the targets of Casp3 which gets cleaved in order to bring about various changes in the cellular environment and to help in completion of the apoptotic process. Both Dffa and Dffb are cleaved by Casp3 to effect the DNA fragmentation^28^ while Gsn cleavage brings about morphological changes to the cell during apoptosis^29^. Ngf^30^ has been previously reported to be closely associated with Casp3. Akt-1 activation in response to cytokine receptor signalling has been associated with anti-apoptotic processes^31^. In our analysis we observed that although Akt-1 is linked with other modules, its association with Caspase-3 is strong, and as a result Akt-1 has been grouped in Module 1. However, the scenario changes drastically once we enhance the network including the primary interactors of each of the proteins in the 1^st^ order interactome model to develop the 2^nd^ order interactome.

The 2^nd^ order interactome segregated into 12 modules, among which 7 were larger groups, each containing 6 or more members while the rest were smaller groups with single nodes (Fig. 1c). The module configurations of the 2^nd^ order interactome clearly indicate that most members of module 3 and 2 are also present in module 2 & 4 of the 1^st^ order interactome, respectively. Module 3 in 2^nd^ order interactome consists of Casp-8 and FADD, key players of the DD and DISC processes. Module 2 is now an integrated assembly formed from nodes of module 1 and 4 of 1^st^ order interactome and consists of proteins taking part in the intermediate stage and the final execution of apoptosis. A closer look at the 2^nd^ order interactome reveals the 4 major groups apart from 2 and 3. Modules 1, 8 and 9 have been built around few of the major anti-apoptotic proteins of the 1^st^ order interactome for example Akt1, Birc 2, Birc3, Traf2 and TNF. We observed that in 1^st^ order interactome TNF and Traf2 were included within module 3 whereas in 2^nd^ order interactome TNF and Traf2 were placed in modules 8 and 9 respectively. TNF has been earlier reported to be involved in activation of apoptotic pathways^32^,^33^. But from our analysis we propose TNF may have some anti-apoptotic function based on its interactions with cytokines IL-1a and IL-6, that have been reported to be involved in cell survival^34^,^35^. The modules 8, 10 and 11 being influenced by the anti-apoptotic proteins form a significant part of this network that was not so prominent in the 1^st^ order interactome. Other modules such as 4, 5, 6, 7 and 12 although consisting of fewer members in the context of our study, have the potential to embark into larger modules if an even bigger network is considered. This is simply because these modules consist of very important proteins that have been known to play pivotal roles in apoptosis.

Degree centrality is simply defined as the interaction score of a particular node within a network. The more interactions a node has within a group of nodes which are mutually interacting among each other, the higher its chance will be to form a module. Hence the community structure formation largely depends upon degree centrality of the nodes within a complex network. Casp3 and FADD were ranked among top 5 proteins when nodes of 1^st^ and 2^nd^ order interactomes were sorted in terms of degree centrality (Table 2). This signifies the pivotal role played by these two proteins in apoptosis and also gives us confidence to interpret the biological significance of modules from graph-theoretic measures. In Table 2 we see an interesting pattern. Nodes in the 1^st^ order interactome that have positive degree centrality scores remained to be in the positive side in the 2^nd^ order interactome. However, degree centrality of nodes that had 0 or negative values in the 1^st^ order connectome either enhanced or got depreciated in 2^nd^ order. In order to explain this pattern we have to carefully analyze both the interactome models. Nodes having positive scores in the 1^st^ order connectome interact not only maximally within their modules but also with other nodes in different modules. Hence, with the increase in number of interacting partners in the 2^nd^ order interactome, the overall connectivity is enhanced for the constituent nodes. For example, Akt1 in the 1^st^ order interactome interacts with several nodes of different modules but not consistently within one module. However, in the 2^nd^ order interactome, the degree centrality of Akt1 increased and creation of a separate module involving Akt1 was observed^36^,^37^. Other nodes like TNF and Traf2 that were in one module in 1^st^ order, increased their interactive partners and gained entry to bigger modules in 2^nd^ order interactome. Nodes that have a predominant role to play in apoptosis maintained their modules and their degree centrality scores across both 1^st^ and 2^nd^ order interactome models.

In conclusion, we have outlined a robust method for studying the interactome underlying apoptosis following CHPV infection. This method may be used to study other metabolic pathways in order to yield important information about the strategic proteins of a specific network and the functionally important modules within the network. In the future, therapeutic targeting of particular proteins in case of various disease conditions needs to be investigated.

## Methods

### Empirical data

In an earlier study^17^, samples of Chandipura Virus was inoculated into Balb/c mouse intraperitoneally (i.p.) post-natal 10 days, at a plaque forming unit (pfu/ml) of 3 × 10^5^. The animals developed CHPV related symptoms of hind limb paralysis, high grade fever and severe weight loss, within 72–96 hours post infection leading to death. From immunoblotting and immunostaining analyses performed on the extracted brain tissue, we found over-expression of 6 proteins of the extrinsic apoptosis pathway: Fas, FADD (Fas-associated Death Domain), Caspase-8, Caspase-3 and XIAP (Poly ADP Ribose Polymerase-1). Our results were further validated using RNAi studies, ELISA assays and flow-cytometric analyses^17^. Table 3 enlists the protein names with their corresponding Uniprot IDs.

### Generation of meta-network

STRING (Search Tool for the Retrieval of Interacting Genes/Proteins) is an open datasource providing information about protein-protein interactions based on experimental data, computational prediction methods and public database^38^. STRING 9.1 database contains information about more than 5.4 million proteins and >1100 organisms^39^. The database has two modes of applications: Protein-mode (for protein interactions) and COG-mode (for gene interactions). STRING imports protein association information from databases of physical interaction and curated biological pathway knowledge (MINT, HPRD, BIND, DIP, BioGRID, KEGG, Reactome, IntAct, EcoCyc, NCI-Nature Pathway Interaction Database, GO). Protein/genes are queried to the STRING database which as an output that represents the associations in the form of a graph network with nodes (proteins/genes) and edges (interactions). The edges are weighted, integrated and a confidence score is assigned to each of them based upon the evidence of the association obtained from experimental data, computational prediction and public data collection methods. Based on these edges are assigned various shades of color (blue)^38^. The prediction methods generally used in determining the interactions are:

### Neighbourhood

This method of prediction utilizes the theory that protein interactions validated in case of one or more species is predicted to carry more weightage and confidence score.

### Gene Fusion

Proteins fused in one genome are likely to be functionally linked and hence carry stronger association.

### Co-occurrence

Occurrence of two proteins within the same metabolic pathway is predicted to functionally linked with each other. Hence their co-occurrence strengthens their confidence score.

### Co-expression

Simultaneous expression of two proteins is also predicted to have strong interaction between them.

## Generation of 1^st^ order interactome

The 5 proteins identified through molecular analyses were queries in the STRING 9.1 that produced 26 interacting partners as an output from the *Mus musculus* database. The STRING 9.1 software defines significance of the interactions between various queried proteins in terms of confidence score. This confidence score is an empirical score defined by the number of citations and experimental evidence for a particular interaction. The highest (0.95) confidence score in the database, that defines the significance of interactions between various queried protein was chosen to extract interactomes in this study. Furthermore, we limited the number of interacting partner to 1000 in the provision for maximum interacting partners using active prediction methods as neighbourhood, gene fusion, co-occurence and co-expression.

## Generation of 2^nd^ order network

In order to investigate the structure of an even larger network we queried for interacting partners of all the 26 proteins obtained from the previous analysis. The 2^nd^ order connectome in Fig. 1b was generated from STRING 9.1 database using same confidence score (0.95) as for the 1^st^ order connectome and limiting to 1000 interacting partners

### Graph theoretic analysis

The adjacency matrices for graph theoretic analysis were created from 1^st^ and 2^nd^ order interactomes. Visual Connectome analysis tool box in MATLAB was used to compute the modularity score and the degree centrality of all the nodes^40^.

### Degree Centrality

Degree centrality is the property that defines the connectivity of particular node with other nodes of the same network. This means the higher number of connections of a particular node with other nodes in a network, higher is its degree centrality. The node with the highest degree centrality is the one through which maximum edges pass.

Degree centrality of a vertex *v*, for a given graph G = (V,E) with |V| vertices and |E| edges is defined as
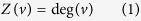


### Modularity

Modularity score is used to measure the community structure within a network. The value of modularity ranges between [−0.5, 1) with 0 and negative values meaning a network with randomly assigned edges to positive values indicating highly communal structure. In a given graph G (V, E) which can be partitioned into two membership variables *s*. If a node *v* falls into community 1 then *s*_*v*_ = 1 or else *s*_*v*_ = −1. An adjacency matrix may be denoted by *A*, which says *A*_*vw*_ = 1 means there is a connection between nodes *v* and *w* and *A*_*vw*_ = 0 when there are no interactions. Modularity (*Q*) is then defined as the fraction of edges that fall within community 1 or 2, minus the expected number of edges within communities 1 and 2 for a random graph with the same node degree distribution as the given graph.

The expected number of edges will be calculated using the concept of Configuration Models^41^. The configuration model is a randomized representation of a particular graph. Given a network with n nodes, where each node *v* has a node degree *k*_*v*_, the configuration model intercepts each edge into two halves, and then each half edge is defined as a stub, that is rewired randomly with any other stub in the network even allowing self loops. Hence even though the node degree distribution of the graph remains intact, the configuration model results in a completely random network. Let the total number of stubs be
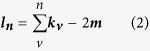
If two nodes *v* and *w* with node degrees *k*_*v*_ and *k*_*w*_, respectively are these nodes, then
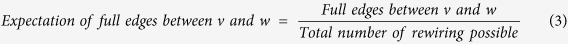

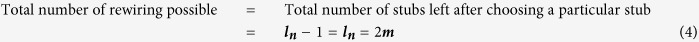

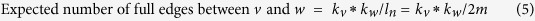
Modularity score is calculated as
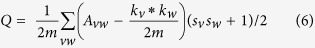
The above equation is valid for two-community structure and can be generalized into *c*-community structure.
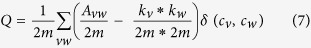


### Modularity Optimization

The (6) can be re-written as:
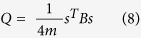
where *s* is column vector whose elements are *s*_*i*_; and *B* is a symmetric matrix
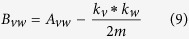
*B*_*vw*_ is also referred to as the modularity matrix which will be having elements whose rows and columns sum upto 0, so that it always has an eigen vector (1, 1, 1..) with eigen value 0^42^. The algorithm that we used, initially divided the network into two communities and in further iterations the community structure is subdivided. For a group *g* of size *n*_*g*_ we can express the contribution to modularity as
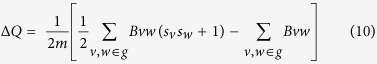
which simplify to: 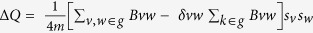 that can be expressed as
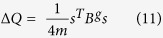
where *δ* stands for Kronecker *δ* symbol and *B*^*g*^ represents the *n*_*g*_ *Xn*_*g*_ matrix with vertices *v, w* in a particular group *g* having values of
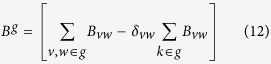
Certainly (8) and (11) are similar and therefore spectral approach^42^ was applied to the generalized modularity matrix to maximize the values of Δ*Q*. 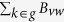 for a complete network happens to be a symmetric matrix and thus (11) turns to nothing but (8). Once Δ*Q* is almost 0 for an indivisible network, then further subdividing beyond this point will not contribute to the increase in modularity value *Q*. This can be used to terminate community structure division.

The algorithm ran with the following theory: The modularity matrix, (9) was constructed for both interactomes and found the most positive eigenvalue and the corresponding eigenvector in each case. The algorithm divided the network into two parts depending upon the signs of the elements of the corresponding vectors, and then subdividing using the generalized modularity matrix (12). In the process Δ*Q* comes to 0 or negative at any stage of subdivision the algorithm left subgraph undivided. Hence, the algorithm would end at a certain point when the optimal network has been estimated. In order to fine tune this method of community structure optimization further, the Visual Connectome toolbox^21^ that we employed uses Kernighan-Lin algorithm^43^.

## Additional Information

**How to cite this article**: Ghosh, S. *et al.* Graph theoretic network analysis reveals protein pathways underlying cell death following neurotropic viral infection. *Sci. Rep.* **5**, 14438; doi: 10.1038/srep14438 (2015).

## Figures and Tables

**Table 1:** Protein names, community structure value (Ci) score of the 1^st^ and 2^nd^ order interactome.

		Module				Module				Module	
Protein Name	Protein	1^st^	2^nd^	Protein Name	Protein	1^st^	2^nd^	Protein Name	Protein	1^st^	2^nd^
Caspase-3	Casp3	1	2	Caspase-7	Casp7	4	2	Cyclin-dependent kinase inhibitor 1A (P21)	Cdkn1a		1
Caspase-8	Casp8	2	3	Direct IAP binding protein with low pI	Diablo	4	2	Forkhead box O4	Foxo4		1
Fas	Fas	2	3	Proto-oncogene tyrosine-protein kinase	Fyn		2	cAMP responsive element binding protein 1	Creb1		10
Fas-associated Death Domain	Fadd	2	3	Cylindromatosis	Cyld		3	Transformed mouse 3T3 cell double minute 2	Mdm2		10
X-linked inhibitor of apoptosis protein	Xiap	4	2	Cluster of Differentiation- 40	Cd40		3	Cyclin-dependent kinase inhibitor 1B	Cdkn1b		1
Fas Ligand	Fasl	2	3	TNF receptor-associated factor 3	Traf3		8	Forkhead box O1	Foxo1		1
Tumor Necrosis Factor (TNF) receptor-associated factor 2	Traf2	3	3	Ubiquitin-c	Ubc		10	Tuberous sclerosis 2	Tsc2		1
Tumor necrosis factor receptor type 1-associated DEATH domain	Tradd	3	3	Toll-like receptor adaptor molecule 1	Ticam1		8	Mechanistic target of rapamycin (serine/threonine kinase)	Mtor		1
Receptor-interacting serine/threonine-protein kinase 1	Ripk1	3	3	Death domain-containing protein	Cradd		3	Phosphatase and tensin homolog	Pten		1
CASP8 and FADD-like apoptosis regulator	Cflar	2	3	Inhibitor of nuclear factor kappa-B kinase subunit gamma	Ikbkg		3	RPTOR independent companion of MTOR, complex 2	Rictor		1
Tumor necrosis factor receptor superfamily, member 10b	Tnfrsf10b	2	3	TNF receptor-associated factor 1	Traf1		8	SMT3 suppressor of mif two 3 homolog 1	Sumo1		6
B-cell receptor associated protein 31	Bcap31	2	3	Toll-like receptor 4	Tlr4		8	Mitogen-activated protein kinase kinase kinase 5	Map3k5		1
Baculoviral IAP repeat-containing protein 2	Birc2	4	8	Mitogen-activated protein kinase kinase kinase 7	map3k7		8	DNA methyltransferase 1-associated protein 1	Dmap1		10
											
Baculoviral IAP repeat-containing protein 3	birc3	4	8	Profilin1	Pfn1		10	Homeodomain interacting protein kinase 1	Hipk1		10
Gelsolin	Gsn	1	10	B cell leukemia/lymphoma 2	Bcl2		1	Alpha thalassemia/mental retardation syndrome X-linked homolog	Atrx		10
DNA fragmentation factor subunit alpha	Dffa	1	2	BCL2-like 11 (apoptosis facilitator)	bcl2l11		1	v-rel avian reticuloendotheliosis viral oncogene homolog A	Rela		9
DNA fragmentation factor subunit beta	Dffb	1	2	Signal transducer and activator of transcription 3	Stat3		8	FBJ osteosarcoma oncogene	Fos		9
Apoptotic protease activating factor 1	Apaf1	4	2	Furin	Furin		10	Adiponectin, C1Q and collagen domain containing	Adipoq		5
Nerve Growth Factor	Ngf	1	11	Transient receptor potential cation channel, subfamily V, member 1	Trpv1		8	Leptin	Lep		9
RAC-alpha serine/threonine-protein kinase	Akt1	1	1	Fibroblast growth factor receptor substrate 2	Frs2		3	Interleukin-6	Il6		9
Death-associated protein 6	Daxx	2	10	Neurotrophic tyrosine kinase, receptor, type 2	Ntrk2		3	Interleukin-1a	Il1a		9
Tumor Necrosis Factor	Tnf	3	9	Nerve growth factor receptor (TNFR superfamily, member 16)	Ngfr		8	Peroxisome proliferative activated receptor, gamma, coactivator 1 alpha	Ppargc1a		7
Tumor necrosis factor receptor superfamily member 1A	tnfrsf1a	3	3	Neurotrophic tyrosine kinase, receptor, type 1	Ntrk1		8	Calspin	Clspn		10
Caspase-9	Casp9	4	2	src homology 2 domain-containing transforming protein C1	Shc1		8				

The protein names for the table were arranged according to the chronology in which they have been queried from the STRING 9.1 database. The first 5 are the proteins whose expressions were monitored empirically the next 21 were the primary interacting partners. The next 45 secondary interacting partners were added to the list.

**Table 2:** The protein names were arranged in decreasing order according to their respective degree centrality (Z) scores for both 1^st^ (a) and 2^nd^ order (b) interactome.

1^st^ Order		2^nd^ Order					
Protein	Z score	Protein	Z score	Protein	Z score	Protein	Z score
Casp3	1.9518	Akt1	3.1326	Ntrk1	0	Ntrk2	−0.4472
Casp8	1.569143	Casp8	2.0533	Shc1	0	Fas	−0.5019
Casp9	1.224745	Ngf	1.7889	Mdm2	0	Fasl	−0.5019
Casp7	1.224745	Fadd	1.7339	Sumo1	0	Tradd	−0.5019
Fadd	0.998545	Casp3	1.6202	Map3k5	0	Tnfrsf10b	−0.5019
Diablo	0.612372	Tnf	1.3618	Dmap1	0	Apaf1	−0.5401
Fas	0.427948	Il6	1.3618	Hipk1	0	Rela	−0.5447
Traf2	0	Ripk1	1.0951	Atrx	0	Lep	−0.5447
Tradd	0	Casp9	0.9001	Adipoq	0	Il1a	−0.5447
Ripk1	0	Birc2	0.7771	Ppargc1a	0	Cdkn1a	−0.7627
Tnf	0	birc3	0.7771	Clspn	0	Foxo4	−0.7627
tnfrsf1a	0	Cd40	0.7771	bcl2l11	−0.0545	Cyld	−0.8213
Fasl	−0.14265	Tlr4	0.7771	Stat3	−0.0545	Traf1	−0.9991
Cflar	−0.14265	Traf2	0.4563	Foxo1	−0.0545	Bcap31	−1.1407
Tnfrsf10b	−0.14265	Tsc2	0.2996	Traf3	−0.111	Cradd	−1.1407
Dffa	−0.24398	Mtor	0.2996	map3k7	−0.111	Creb1	−1.1802
Dffb	−0.24398	Xiap	0.18	Cflar	−0.1825	Dffa	−1.2601
Ngf	−0.24398	Casp7	0.18	Ikbkg	−0.1825	Dffb	−1.2601
Akt1	−0.24398	Diablo	0.18	Fyn	−0.4086	Ticam1	−1.8872
Xiap	−0.61237	tnfrsf1a	0.1369	Bcl2	−0.4086		
Birc2	−0.61237	Fos	0.0908	Cdkn1b	−0.4086		
birc3	−0.61237	Gsn	0	Pten	−0.4086		
Gsn	−0.9759	Daxx	0	Rictor	−0.4086		
Apaf1	−1.22474	Ubc	0	Furin	−0.4472		
Bcap31	−1.28384	Pfn1	0	Trpv1	−0.4472		
Daxx	−1.28384	Ngfr	0	Frs2	−0.4472		

**Table 3:** The table enlists the Uniprot identification numbers for all the proteins which were used in our analysis.

Protein Symbol	UniProt ID	Protein Symbol	Uniprot ID
Casp3	P70677	map3k7	Q923A8
Casp8	O89110	Pfn1	P62962
Fas	P25446	Bcl2	P10417
Fadd	Q61160	bcl2l11	O54918
Xiap	Q60989	Stat3	P42227
Fasl	P41047	Furin	P23188
Traf2	P39429	Trpv1	Q704Y3
Tradd	Q3U0V2	Frs2	Q8C180
Ripk1	Q60855	Ntrk2	P15209
Cflar	O35732	Ngfr	Q8CFT3
Tnfrsf10b	Q9QZM4	Ntrk1	Q3UFB7
Bcap31	Q61335	Shc1	P98083
Birc2	Q62210	Cdkn1a	P39689
birc3	O08863	Foxo4	Q9WVH3
Gsn	P13020	Creb1	Q01147
Dffa	O54786	Mdm2	P23804
Dffb	O54788	Cdkn1b	P46414
Apaf1	O88879	Foxo1	Q9R1E0
Ngf	P01139	Tsc2	Q7TT21
Akt1	P31750	Mtor	Q9JLN9
Daxx	O35613	Pten	O08586
Tnf	P06804	Rictor	Q6QI06
tnfrsf1a	P25118	Sumo1	P63166
Casp9	Q8C3Q9	Map3k5	Q14AY4
Casp7	P97864	Dmap1	Q9JI44
Diablo	Q9JIQ3	Hipk1	O88904
Fyn	P39688	Atrx	Q61687
Cyld	Q80TQ2	Rela	Q04207
Cd40	P27512	Fos	P01101
Traf3	Q60803	Adipoq	Q60994
Ubc	P0CG50	Lep	P41160
Ticam1	Q80UF7	Il6	P08505
Cradd	O88843	Il1a	P01582
Ikbkg	Q8VC91	Ppargc1a	O70343
Traf1	P39428	Clspn	Q80YR7
Tlr4	Q9QUK6		

## References

[b1] Beltrao P., Bork P., Krogan N. J. & van Noort V. Evolution and functional cross-talk of protein post-translational modifications. Mol Syst Biol 9, 714 (2013). 24366814 10.1002/msb.201304521 PMC4019982

[b2] Kim J. & Copley S. D. Inhibitory cross-talk upon introduction of a new metabolic pathway into an existing metabolic network. Proc Natl Acad Sci USA 109, E2856–2864 (2012). 22984162 10.1073/pnas.1208509109 PMC3479479

[b3] Cusick M. E., Klitgord N., Vidal M. & Hill D. E. Interactome: gateway into systems biology. Hum Mol Genet 14 Spec No. 2, R171–181 (2005). 16162640 10.1093/hmg/ddi335

[b4] Rual J. F. *et al.* Towards a proteome-scale map of the human protein-protein interaction network. Nature 437, 1173–1178 (2005). 16189514 10.1038/nature04209

[b5] Schrattenholz A., Groebe K. & Soskic V. Systems biology approaches and tools for analysis of interactomes and multi-target drugs. Methods Mol Biol 662, 29–58 (2010). 20824465 10.1007/978-1-60761-800-3_2

[b6] Meyer M. J., Das J., Wang X. & Yu H. INstruct: a database of high-quality 3D structurally resolved protein interactome networks. Bioinformatics 29, 1577–1579 (2013). 23599502 10.1093/bioinformatics/btt181 PMC3673217

[b7] Stojmirovic A. & Yu Y. K. Information flow in interaction networks. J Comput Biol 14, 1115–1143 (2007). 17985991 10.1089/cmb.2007.0069

[b8] Mason O. & Verwoerd M. Graph theory and networks in Biology. IET Syst Biol 1, 89–119 (2007). 17441552 10.1049/iet-syb:20060038

[b9] Christensen C., Thakar J. & Albert R. Systems-level insights into cellular regulation: inferring, analysing, and modelling intracellular networks. IET Syst Biol 1, 61–77 (2007). 17441550 10.1049/iet-syb:20060071

[b10] Chang X., Xu T., Li Y. & Wang K. Dynamic modular architecture of protein-protein interaction networks beyond the dichotomy of ‘date’ and ‘party’ hubs. Sci Rep 3, 1691 (2013). 23603706 10.1038/srep01691 PMC3631766

[b11] Rubinov M. & Sporns O. Weight-conserving characterization of complex functional brain networks. Neuroimage 56, 2068–2079 (2011). 21459148 10.1016/j.neuroimage.2011.03.069

[b12] Ding S. L. & Rockland K. S. Modular organization of the monkey presubiculum. Exp Brain Res 139, 255–265 (2001). 11545464 10.1007/s002210100778

[b13] Valencia M. *et al.* Complex modular structure of large-scale brain networks. Chaos 19, 023119 (2009). 19566254 10.1063/1.3129783

[b14] Song J. & Singh M. From hub proteins to hub modules: the relationship between essentiality and centrality in the yeast interactome at different scales of organization. PLoS Comput Biol 9, e1002910 (2013). 23436988 10.1371/journal.pcbi.1002910 PMC3578755

[b15] Bhatt P. N. & Rodrigues F. M. Chandipura: a new Arbovirus isolated in India from patients with febrile illness. Indian J Med Res 55, 1295–1305 (1967). 4970067

[b16] Chadha M. S. *et al.* An outbreak of Chandipura virus encephalitis in the eastern districts of Gujarat state, India. Am J Trop Med Hyg 73, 566–570 (2005). 16172482

[b17] Ghosh S., Dutta K. & Basu A. Chandipura virus induces neuronal death through Fas-mediated extrinsic apoptotic pathway. J Virol 87, 12398–12406 (2013). 24027318 10.1128/JVI.01864-13 PMC3807914

[b18] Tandale B. V. *et al.* Chandipura virus: a major cause of acute encephalitis in children in North Telangana, Andhra Pradesh, India. J Med Virol 80, 118–124 (2008). 18041027 10.1002/jmv.21041

[b19] Balakrishnan A. & Mishra A. C. Immune response during acute Chandipura viral infection in experimentally infected susceptible mice. Virol J 5, 121 (2008). 18937835 10.1186/1743-422X-5-121 PMC2577095

[b20] Mavale M. S. *et al.* Experimental transmission of Chandipura virus by Phlebotomus argentipes (diptera: psychodidae). Am J Trop Med Hyg 76, 307–309 (2007). 17297040

[b21] Rubinov M. & Sporns O. Complex network measures of brain connectivity: uses and interpretations. Neuroimage 52, 1059–1069 (2010). 19819337 10.1016/j.neuroimage.2009.10.003

[b22] Park H. H. *et al.* The death domain superfamily in intracellular signaling of apoptosis and inflammation. Annu Rev Immunol 25, 561–586 (2007). 17201679 10.1146/annurev.immunol.25.022106.141656 PMC2904440

[b23] Valmiki M. G. & Ramos J. W. Death effector domain-containing proteins. Cell Mol Life Sci 66, 814–830 (2009). 18989622 10.1007/s00018-008-8489-0 PMC11131443

[b24] Pennarun B. *et al.* Playing the DISC: turning on TRAIL death receptor-mediated apoptosis in cancer. Biochim Biophys Acta 1805, 123–140 (2010). 19961901 10.1016/j.bbcan.2009.11.004

[b25] Kim J. W., Choi E. J. & Joe C. O. Activation of death-inducing signaling complex (DISC) by pro-apoptotic C-terminal fragment of RIP. Oncogene 19, 4491–4499 (2000). 11002422 10.1038/sj.onc.1203796

[b26] Tan B. M. *et al.* Baculoviral inhibitors of apoptosis repeat containing (BIRC) proteins fine-tune TNF-induced nuclear factor kappaB and c-Jun N-terminal kinase signalling in mouse pancreatic beta cells. Diabetologia 56, 520–532 (2013). 23250032 10.1007/s00125-012-2784-x

[b27] Wang Y. *et al.* Gene network revealed involvements of Birc2, Birc3 and Tnfrsf1a in anti-apoptosis of injured peripheral nerves. PLoS One 7, e43436 (2012). 23028454 10.1371/journal.pone.0043436 PMC3444457

[b28] Nordstrom E. A. *et al.* A human-specific role of cell death-inducing DFFA (DNA fragmentation factor-alpha)-like effector A (CIDEA) in adipocyte lipolysis and obesity. Diabetes 54, 1726–1734 (2005). 15919794 10.2337/diabetes.54.6.1726

[b29] Harms C. *et al.* Neuronal gelsolin prevents apoptosis by enhancing actin depolymerization. Mol Cell Neurosci 25, 69–82 (2004). 14962741 10.1016/j.mcn.2003.09.012

[b30] Holub J. L., Qiu Y. Y., Chu F. & Madonna M. B. The role of nerve growth factor in caspase-dependent apoptosis in human BE(2)C neuroblastoma. J Pediatr Surg 46, 1191–1196 (2011). 21683221 10.1016/j.jpedsurg.2011.03.054

[b31] Green B. D. *et al.* Akt1 is the principal Akt isoform regulating apoptosis in limiting cytokine concentrations. Cell Death Differ 20, 1341–1349 (2013). 23787999 10.1038/cdd.2013.63 PMC3770321

[b32] Fiers W. *et al.* TNF-induced intracellular signaling leading to gene induction or to cytotoxicity by necrosis or by apoptosis. J Inflamm 47, 67–75 (1995). 8913931

[b33] Rath P. C. & Aggarwal B. B. TNF-induced signaling in apoptosis. J Clin Immunol 19, 350–364 (1999). 10634209 10.1023/a:1020546615229

[b34] Kast R. E. & Altschuler E. L. Anti-apoptosis function of TNF-alpha in chronic lymphocytic leukemia: lessons from Crohn’s disease and the therapeutic potential of bupropion to lower TNF-alpha. Arch Immunol Ther Exp (Warsz) 53, 143–147 (2005). 15928583

[b35] Seifert J. K. *et al.* Large volume hepatic freezing: association with significant release of the cytokines interleukin-6 and tumor necrosis factor a in a rat model. World J Surg 26, 1333–1341 (2002). 12297923 10.1007/s00268-002-6139-5

[b36] Chang H. Y. *et al.* Activation of apoptosis signal-regulating kinase 1 (ASK1) by the adapter protein Daxx. Science 281, 1860–1863 (1998). 9743501 10.1126/science.281.5384.1860

[b37] Hatai T. *et al.* Execution of apoptosis signal-regulating kinase 1 (ASK1)-induced apoptosis by the mitochondria-dependent caspase activation. J Biol Chem 275, 26576–26581 (2000). 10849426 10.1074/jbc.M003412200

[b38] von Mering C. *et al.* STRING: a database of predicted functional associations between proteins. Nucleic Acids Res 31, 258–261 (2003). 12519996 10.1093/nar/gkg034 PMC165481

[b39] Franceschini A. *et al.* STRING v9.1: protein-protein interaction networks, with increased coverage and integration. Nucleic Acids Res 41, D808–815 (2013). 23203871 10.1093/nar/gks1094 PMC3531103

[b40] Dai D. & Huiguang H. VisualConnectome: Toolbox for brain network visualization and analysis (Abstract). Human Brain Mapping (2011).

[b41] Pereira T. *et al.* Connectivity-driven coherence in complex networks. Phys Rev Lett 110, 234103 (2013). 25167497 10.1103/PhysRevLett.110.234103

[b42] Newman M. E. Modularity and community structure in networks. Proc Natl Acad Sci USA 103, 8577–8582 (2006). 16723398 10.1073/pnas.0601602103 PMC1482622

[b43] Wernisch L., Hunting M. & Wodak S. J. Identification of structural domains in proteins by a graph heuristic. Proteins 35, 338–352 (1999). 10328269

